# S-palmitoylation: a novel insight in the development and immunotherapy of oral squamous cell carcinoma

**DOI:** 10.7150/jca.110721

**Published:** 2025-06-12

**Authors:** Xue-ting Yuan, Jing-ru Wang, Ying Yang, Jian-gang Ren

**Affiliations:** 1 State Key Laboratory of Oral & Maxillofacial Reconstruction and Regeneration, Key Laboratory of Oral Biomedicine Ministry of Education, Hubei Key Laboratory of Stomatology, School & Hospital of Stomatology, Wuhan University, Wuhan, 430079, China.; 2 Xianning Medical College, Hubei University of Science and Technology, Xianning, China.; 3 Department of Oral and Maxillofacial Surgery, School and Hospital of Stomatology, Wuhan University, Wuhan, 430079, China.

**Keywords:** OSCC, immunotherapy, palmitoylation, palmitoyltransferase, signaling pathway

## Abstract

S-palmitoylation (hereinafter referred to as palmitoylation) is a reversible lipid modification that has recently received considerable attention in cancer research. Despite its known association with tumour progression and treatment response, it remains unclear how palmitoylation could be targeted for enhancing therapeutic outcomes in oral squamous cell carcinoma (OSCC). This review summarises palmitoylated proteins common in various cancers and highlights emerging targets specific to OSCC, emphasising their roles in protein stability, signalling pathways, and cellular behaviour. Additionally, we explore new trends in targeting palmitoylated proteins to manage cancer progression and bolster the immune response in OSCC. Furthermore, this review highlights existing knowledge gaps and calls for detailed investigations into OSCC-specific palmitoylation mechanisms, including the expression levels of palmitoylated proteins and palmitoylation enzymes and their effect on OSCC signalling pathways.

## 1. Introduction

Oral squamous cell carcinoma (OSCC) is the most common subtype of head and neck cancer. Owing to its aggressive nature and propensity for lymph node metastasis, the overall five-year survival rate is less than 60%, with many patients experiencing rapid recurrence 1.

S-palmitoylation (hereinafter referred to as palmitoylation) is a reversible lipid-based post-translational modification implicated in various human diseases 2, 3. This modification involves the covalent attachment of the 16-carbon fatty acid, palmitate, to cysteine (Cys) residues on protein side chains via a labile thioester bond 4. Protein palmitoylation is governed by the enzymatic actions of palmitoyltransferases and depalmitoylases. To date, the majority of identified palmitoyltransferases are members of the zinc finger DHHC-type containing (ZDHHC) palmitoyltransferase family 5. S-palmitoylation is also modulated by depalmitoylases, including proteins 17A/B/C with α/β hydrolase domain (ABHD17A/B/C) and ABHD10, palmitoyl-protein thioesterase 1/2 (PPT1/2), and acyl protein thioesterase 1/2 (APT1/2 or LYPLA1/2) 4.

Palmitoylation of key oncogenes and tumour suppressors is closely associated with cancer development and progression 6-8. For instance, palmitoylation of proteins such as epidermal growth factor receptor (EGFR) and members of the RAS family, which are prevalent in many cancers 9-11, as well as palmitoylation of RAB27 proteins, recently implicated in OSCC 12, has been shown to influence the progression of OSCC in various ways. In addition, studies targeting palmitoylation at an immune checkpoint have recently attracted considerable attention. It has been shown that the palmitoylation of programmed cell death ligand 1 (PD-L1) and interferon gamma receptor 1 (IFNGR1) can affect CD8^+^ T cell infiltration and influence the efficacy of immunotherapy 13. Emerging research indicates that palmitoylation regulates the stability and activation of important proteins, such as gasdermin D, the executor of cellular pyroptosis, and the mitochondrial protein carnitine palmitoyltransferase 1A, affecting tumour immunity 14, 15. These findings underscore the substantial potential of palmitoylation in immunopathology and immunotherapy.

While recent reviews have summarised the molecular functions of protein palmitoylation in various cancers, a thorough evaluation of its role in OSCC remains lacking. We defined our search as the selection of articles with experimental data, reviews, and recent publications from the leading biomedical database, PubMed. Our search queries incorporated synonyms for “palmitoylation” and “oral squamous cell carcinoma.” This review offers a novel perspective on palmitoylation, summarizing recent findings on its impact on key cancer hallmarks in OSCC, its influence on tumor progression and prognosis, and potential therapeutic targets yet to be fully explored. Special emphasis is placed on the transformative role of palmitoylation in immunotherapy.

## 2. Protein Palmitoylation and Tumour Progression in OSCC

### 2.1. SRC family

The SRC family of kinases (SFKs) constitutes a subset of overexpressed tyrosine kinases observed in various cancers, encompassing at least eight closely related proteins: SRC, LYN, FYN, YES, FGR, HCK, LCK, and BLK 16, 17. The distribution of these kinases varies, with SRC, YES, LYN, and FYN found broadly across numerous cell types, whereas BLK, FGR, HCK, and LCK exhibit more restricted expression profiles, predominantly within haematopoietic cells 17.

SFKs are pivotal in cellular signalling and are activated in response to a diverse array of stimuli such as growth factors and adhesion molecules. Once activated, they play an integral role in orchestrating a broad spectrum of cellular processes in the plasma membrane, including cell proliferation, differentiation, migration, and morphological alterations 16.

Research conducted in 2008 highlighted the critical role of palmitoylation in directing cellular SFK trafficking. Specifically, LYN and YES, which undergo monopalmitoylation, are transported to the plasma membrane via the Golgi apparatus as part of the secretory pathway. Conversely, a significant proportion of FYN, which is dually palmitoylated, directly targets the plasma membrane 17.

Palmitoylation enhances LYN's membrane affinity by augmenting its hydrophobicity, thereby promoting its localisation and accumulation within functional membrane domains to stabilise cellular signalling 18. Wheeler et al. further demonstrated that LYN critically drives tumour progression in EGFRvIII-expressing OSCCs. Mechanistically, LYN inhibition in these cells markedly attenuates MAPK phosphorylation and significantly reduces their proliferative, migratory, and invasive capacities 19. Notably, experimental approaches targeting LYN palmitoylation to control OSCC progression remain unexplored to date. Additionally, LCK has been implicated as a potential driver of invasiveness and metastasis in OSCC 20. Inhibition of LCK palmitoylation interrupts early FAS signalling cascades and undermines FAS-mediated apoptosis. Specific knockdown of the palmitoyltransferase ZDHHC21 abrogates LCK activation and its subsequent downstream signalling following FAS receptor activation 21. Collectively, these insights underscore the potential of SFKs inhibition as a therapeutic strategy for patients with OSCC.

### 2.2. Hippo pathway and Yes-associated protein (YAP)/TAZ-TEAD complex

The Hippo signalling pathway orchestrates an array of biological functions such as cell proliferation, self-renewal, differentiation, apoptosis, and organ size regulation by integrating intracellular and extracellular cues 22. Recent comprehensive molecular analyses have elucidated that perturbations within the Hippo pathway and aberrant activity of the downstream effector YAP and transcriptional coactivator with PDZ-binding motif (TAZ) act as prominent oncogenic elements across various cancer types, including OSCC 22.

The aberrantly activated YAP/TAZ-transcriptional enhanced associate domain (TEAD) complex has been implicated in oncogenic processes through a multitude of mechanisms. This complex can dysregulate transcription factors involved in cell cycle regulation, such as activator protein 1 (AP-1), thereby promoting abnormal cellular proliferation, migratory behaviour, and metastasis, and driving AP-1-dependent transcriptional programs essential for cell cycle control in several cancer cell lines 23. Furthermore, hyperactivated YAP/TAZ is responsible for upregulating metabolic gene transcription, leading to increased glucose uptake and transport of high-affinity amino acids, which play an instrumental role in early development by facilitating the epithelial-to-mesenchymal transition (EMT), a process commonly involved in the early stages of oncogenesis 24. Elevated activity of YAP/TAZ is particularly noteworthy in various squamous carcinomas, as they are among the malignancies bearing the highest amplification frequency of these factors, which include cervical, lung, head and neck, bladder, and esophageal squamous cell carcinomas (SCCs) 25. TEAD4, a member of the TEAD transcription factor family, exhibits a critical role, as its knockout enhances YAP phosphorylation and diminishes YAP nuclear localization 26. Furthermore, analysis of TCGA datasets and clinical evaluations reveal an association between elevated TEAD4 expression and various adverse clinical outcomes in OSCC patients, such as higher pathological grades and reduced overall survival 27.

**Figure 1:**
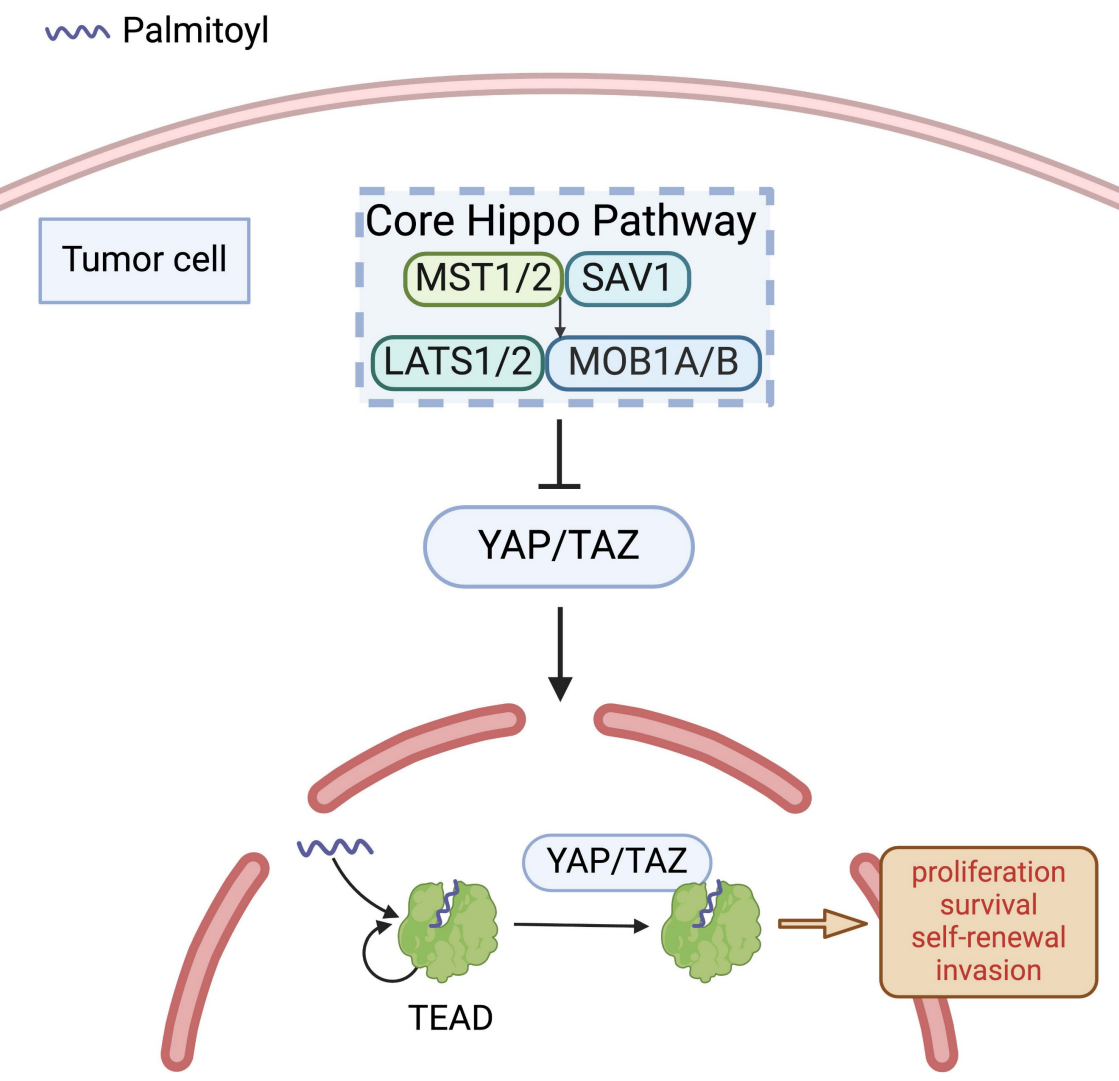
** Palmitoylation of TEAD.** In the absence of Hippo-stimulating signals, the core Hippo kinase cascade is inactive and YES-associated protein (YAP)/Transcriptional coactivator with PDZ-binding motif (TAZ) translocate into the nucleus to associate with YAP/TAZ-transcriptional enhanced associate domain (TEAD) family DNA-binding proteins and co-activate transcriptional programs. TEAD autopalmitoylation is necessary for both YAP/TAZ:TEAD complex formation and YAP/TAZ-mediated transcriptional co-activation.

Upon binding to their coactivators YAP and TAZ, TEAD transcription factors regulate the transcription of target genes within the Hippo pathway 28. The complex formation of YAP/TAZ with TEAD is predicated on an autopalmitoylation reaction in TEAD 22, which is not altering TEAD's localisation but vital for its association with YAP/TAZ (Figure 1), and critically enhancing the transcriptional activity of the complex 28, 29. Notably, high expression activity of the complex enhances TGF-β1-induced EMT by amplifying crosstalk between Hippo and TGF-β/SMAD signalling 30. Mechanistically, TGF-β1-activated SMAD2/3 translocates to the nucleus and cooperates with the YAP/TAZ-TEAD complex to upregulate EMT-promoting transcription factors, while concurrently suppressing epithelial markers 31. This dual regulation fosters cytoskeletal remodelling, extracellular matrix degradation, and metastatic dissemination, thereby driving malignant tumour progression. Conversely, diminished expression activity of the TEAD-YAP complex in OSCC cells contributes to a coordinated suppression of cyclins and cyclin-dependent kinases (CDKs), coupled with enhanced expression of CDK inhibitors. Furthermore, the complex in the nuclei was related closely to transcriptions of G1 arrest-related genes, ultimately leading to reduced cellular proliferation due to cell-cycle arrest in the G1 phase 26.

### 2.3. RAB proteins

RAS-associated binding (RAB) proteins belong to the GTPase Ras superfamily. RAB GTPases play pivotal roles in orchestrating intracellular membrane trafficking and cytoskeletal dynamics, thereby ensuring cellular homeostasis and facilitating a multitude of cellular functions 32. To date, approximately 70 members of this family have been identified in the human genome, with each RAB GTPase localised to distinct membrane compartments that govern the specificity and directionality of membrane transportation processes 32. Moreover, alterations in RAB GTPase expression have been implicated in various cancer-related phenomena including invasion, migration, metabolism, autophagy, exosome secretion, and drug resistance 32.

RAB27, a subset of this protein family, has two isoforms, RAB27A and RAB27B. These proteins are integral to vesicle exocytosis and the release of exosomes, which are critical processes for modulating the tumour microenvironment 33. Notably, RAB27 has been implicated in the progression of various cancers, including breast, melanoma, and colorectal 33-35.

Huang et al. demonstrated that RAB27A enhances EGFR membrane retention through ZDHHC13-mediated palmitoylation, which is critical for EGFR stability and downstream signalling 12. Specifically, RAB27A facilitates the interaction between the palmitoyltransferase ZDHHC13 and EGFR, promoting the covalent attachment of palmitate to EGFR. This palmitoylation event anchors EGFR to the plasma membrane, thereby sustaining its activation and subsequent signalling cascades that drive tumour migration and invasion. Clinically, elevated RAB27A expression was strongly correlated with advanced lymph node metastasis and reduced survival rates in OSCC patients, underscoring its prognostic relevance 12.

Although other members of the RAB family have been studied in various cancers, the specific effects of palmitoylation on these proteins, particularly in OSCC, warrant further investigation.

### 2.4. EGFR

EGFR is a transmembrane receptor tyrosine kinase that relays extracellular signals to intracellular signalling pathways and plays a critical role in cellular communication and function. Overexpression of the EGFR and/or its ligands has been observed in most OSCCs 36. EGFR expression is a well-established adverse prognostic indicator of treatment outcomes and mortality in patients with OSCC 37. Investigations into the role of EGFR signalling have elucidated a dual function in regulating both cellular proliferation and EMT, with the latter being facilitated through extracellular signal-regulated kinase (ERK)1/2 activation 38.

**Figure 2:**
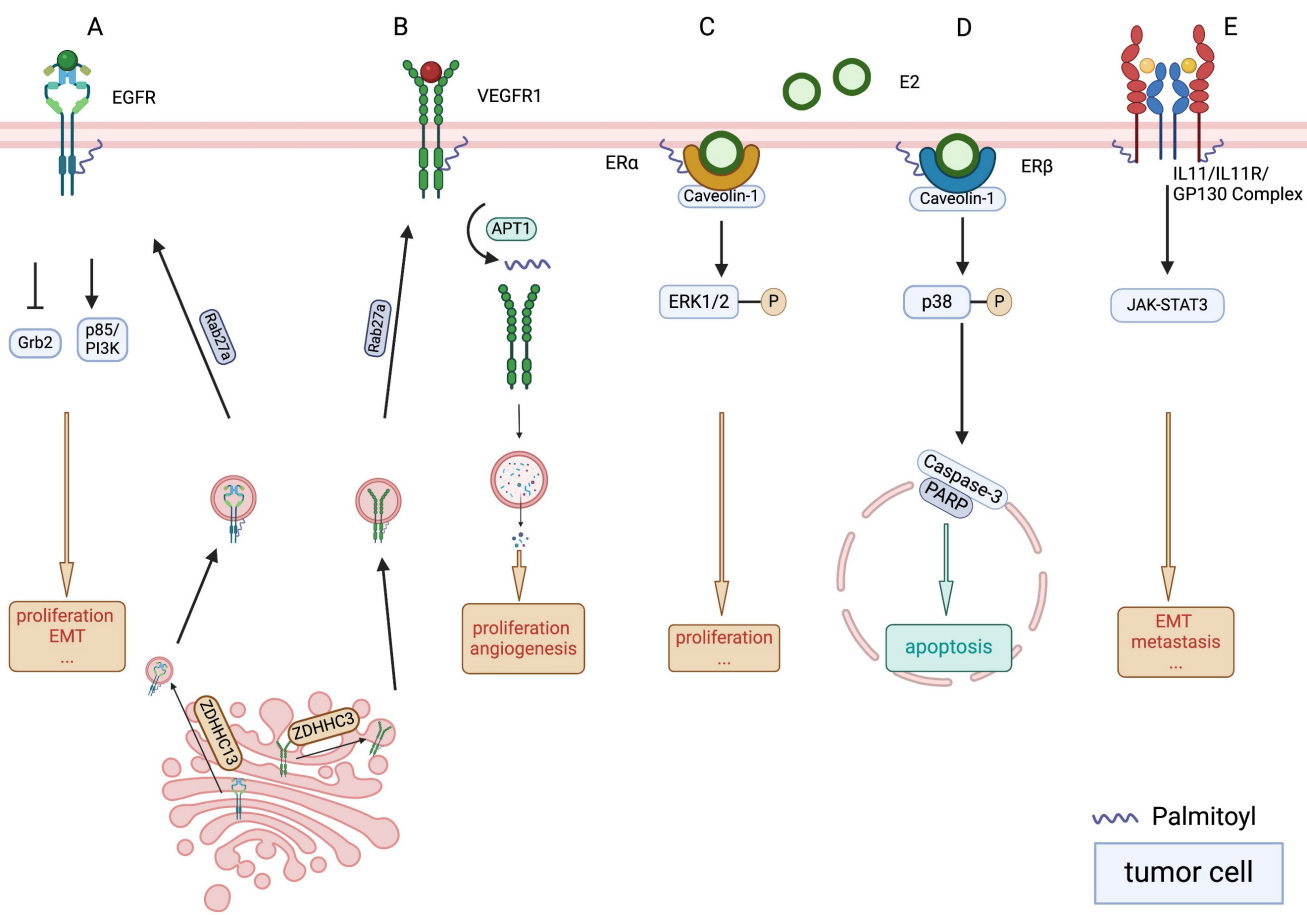
** Palmitoylation of EGFR, VEGFR, ER and GP130.** A. Palmitoylation of the epidermal growth factor receptor (EGFR) results in a decreased association with the adaptor protein Grb2, which plays a pivotal role in mediating mitogen-activated protein kinase (MAPK) signalling. Conversely, there is an increase in the association between palmitoylated EGFR and p85, the regulatory subunit crucial for the phosphoinositide 3-kinase (PI3K) signalling pathway. Additionally, ras-related protein rab-27A (RAB27A) is posited to regulate zinc finger DHHC-type containing (ZDHHC)13, potentially influencing the palmitoylation process of EGFR. B. The stability of vascular endothelial growth factor receptor 1 (VEGFR1) is maintained through the actions of palmitoyltransferase ZDHHC3 and depalmitoylase acyl protein thioesterase (APT1), which serve as critical modulators. A deficiency in palmitoylation escalates the trafficking of VEGFR1 to lysosomes, leading to its degradation and affecting vascular morphogenesis. Furthermore, RAB27A is delineated as an upstream regulator in the palmitoylation process of VEGFR1. C. The palmitoylation of oestrogen receptor alpha (ERα) facilitates its association with the plasma membrane and its interaction with caveolin-1, a membrane protein, which is instrumental in triggering nongenomic activities, including the activation of various signalling pathways and promotion of cell proliferation. D. The localisation of ERβ at the plasma membrane and its interaction with caveolin-1 are contingent upon its palmitoylation. The ERβ-oestradiol (E2) complex's association with caveolin-1 is pivotal for the activation of the p38 mitogen-activated protein kinase pathway, subsequently initiating a pro-apoptotic cascade that includes the activation of caspase-3 and the cleavage of poly (ADP-ribose) polymerase. E. Interleukin 11 (IL-11) activates JAK-STAT3 signalling via the IL-11 receptor alpha subunit/glycoprotein 130 (GP130) receptors, subsequently enhance tumour metastasis. Palmitoylation of GP130 mediated its localisation on the membrane surface.

Recent research has revealed the oncogenic potential of palmitoylated EGFR. Kadry et al. suggested that palmitoylation of EGFR might lead to selective signalling responses specific to various ligands 39. Palmitoylation reduces the association of the adaptor protein growth factor receptor-bound protein 2, which mediates mitogen-activated protein kinase (MAPK) signalling, while enhancing its interaction with p85, the regulatory subunit of the PI3K signalling complex (Figure 2A). This modification is achieved by increasing the association of the C-terminal domain with the plasma membrane 39. Palmitoylation of EGFR plays a critical role in receptor turnover. Increased palmitoylation of EGFR elevates overall receptor levels and reduces lysosomal trafficking, significantly affecting signal activation when palmitoylation of a small receptor pool is inhibited 39. Moreover, EGFR localises to the mitochondria. The activation of mitochondrial EGFR (mtEGFR) by epidermal growth factor (EGF) triggers the synthesis of new palmitate 40. This newly synthesised palmitate activates mtEGFR via palmitoylation, which subsequently promotes mitochondrial fusion and cell survival, thereby aiding cancer progression 40.

Guo et al. discovered that EGFR palmitoylation, mediated by ZDHHC13, plays a crucial role in EGFR localization, where ADP-ribosylation factor 6 (ARF6) serves as an essential component 41. N-myristoylated ARF6 engages with palmitoylated EGFR through lipid-lipid interactions, which then attracts the exocyst complex, aiding in the budding of EGFR from the Golgi apparatus and promoting its transport to the plasma membrane in the GTP-bound state 41. The intracellular activation of EGFR occurs via fatty acid synthase-dependent palmitoylation. Inhibition of fatty acid synthase or palmitoyltransferase diminishes EGFR activity, leading to a reduction in EGFR levels, rendering cancer cells more responsive to EGFR tyrosine kinase inhibitors 42. Runkle et al. discovered that inhibition of ZDHHC20-mediated EGFR palmitoylation increases EGF-induced EGFR activation for the survival of cancer cells. Loss of palmitoylation prolongs EGFR signal activation and increases cell sensitivity to inhibition by EGFR tyrosine kinase inhibitors 43. Furthermore, the necessity of EGFR palmitoylation for the PI3K-AKT-MYC signalling pathway in KRAS-mutant lung adenocarcinoma has been established. Silencing the ZDHHC20 gene reduces PI3K-AKT signalling and MYC expression while increasing sensitivity of tumour cells to PI3K inhibitors, identifying ZDHHC20 inhibition as a potential therapeutic target within the PI3K-AKT axis 44. Taken together, combining ZDHHC20-targeted therapies (e.g., palmitoyltransferase inhibitors or specific competing peptides) with EGFR tyrosine kinase inhibitors or PI3K inhibitors may synergistically overcome treatment resistance in OSCC.

Different palmitoyltransferases may be expressed at different levels and have different effects. Therefore, the therapeutic deployment of palmitoyltransferase antagonists in the context of OSCC requires additional comprehensive investigation.

### 2.5. VEGFR

The vascular endothelial growth factor (VEGF) family and its cognate tyrosine kinase receptors (VEGFRs) are pivotal regulators of angiogenesis, playing an essential role in this process. Angiogenesis is critical not only for physiological vascular development but also for tumour progression and has been implicated as the principal facilitator of tumour vascularization 45. This is relevant because enhanced angiogenesis is closely associated with accelerated tumour invasion and metastasis. According to observations by Mărgăritescu et al., VEGF is expressed in a significant majority (87%) of OSCC specimens, affirming its prevalence within these tumours 46. Tong et al. elucidated the biphasic role of VEGF in the pathogenesis of OSCC; it is both proangiogenic and pro-tumourigenic 47. Suppression of either VEGFRs or its specific ligands results in a decrease in OSCC cell proliferation, indicating that these factors are critical for cancer cell growth 48.

Recent research has shifted focus to targeting VEGFR palmitoylation as an innovative research frontier. A link between palmitoylation of VEGFR1 and its subsequent stability and turnover has been reported. VEGFR1 is an endothelial cell-specific decoy receptor that inherently counteracts blood vessel morphogenesis. Investigations conducted by Joshua et al. underscored the regulatory role of palmitoylation in VEGFR1, which functions as a molecular switch to govern its stability and endocytic trafficking (Figure 2B). They identified palmitoyltransferase ZDHHC3 and depalmitoylase APT1 as critical modulators of this process 49. Moreover, their research pinpointed RAB27A is an upstream regulator of VEGFR1 palmitoylation. RAB27A deficiency disrupts proper palmitoylation of VEGFR1, leading to augmented trafficking of the receptor to lysosomes for degradation, thereby disturbing vascular morphogenesis both in vitro and in vivo 49. Research into OSCC cell models has demonstrated robust inhibition of cell growth when exposed to VEGFR inhibitors 50. These findings suggest that the modulation of VEGFR1 palmitoylation may represent a potential avenue for therapeutic intervention in pathological angiogenesis of OSCC.

### 2.6. PD-L1

In the canonical PD-L1/ programmed cell death protein 1 (PD-1) signalling pathway, PD-L1 impedes lymphocyte function by interacting with PD-1 on the surface of tumour-infiltrating lymphocytes 51, which will be elaborated later in section 3. In addition to the well-known cell-extrinsic interactions, tumour cell-intrinsic PD-L1 signalling plays a pivotal role in tumourigenesis and resistance to therapies.

**Figure 3:**
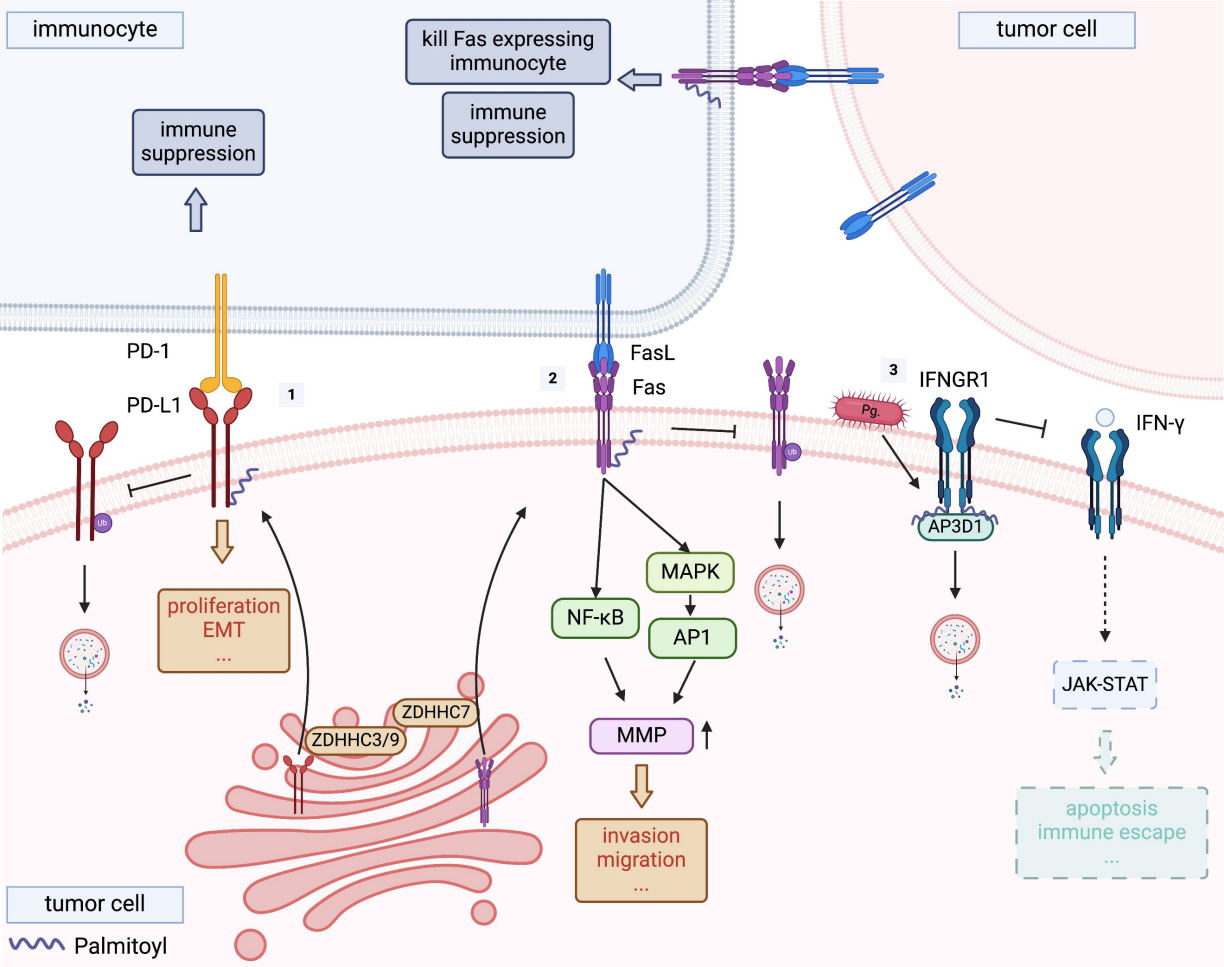
** Palmitoylation of PD-L1, FAS and IFNGR1.** 1 Programmed cell death ligand 1 (PD-L1) is palmitoylated by ZDHHC3 or ZDHHC9, and stabilises PD-L1 by blocking its ubiquitination, consequently suppressing PD-L1 degradation by lysosomes. 2 ZDHHC7 was the main FAS palmitoyltransferase, demonstrating that ZDHHC7-mediated palmitoylation protects FAS from lysosomal degradation and allow a proper localization, thereby regulating its expression. 3 Palmitoylated interferon gamma receptor 1 (IFNGR1) is sorted by adaptor protein adaptor-related protein complex 3 subunit delta 1 (AP3D1) to lysosomes for degradation, driving tumour immune escape. Porphyromonas gingivalis infection promotes this process.

Tumour-intrinsic PD-L1 signalling involves the activation of cellular functions triggered by PD-L1 located on the cell surface, within the cytosol, or in the nucleus 52. These signals precipitate a myriad of cell-intrinsic biological consequences, including regulation of tumour growth, survival pathways, cellular stemness, immune modulation, responses to DNA damage, and gene expression regulation (Figure 3-1). Notably, many of these effects are independent of the PD1 interaction 52. In hypopharyngeal squamous cell carcinoma, PD-L1 enhances the proliferation, migration, and invasion capabilities of FaDu cells (a cell line derived from a hypopharyngeal tumour exhibiting epithelial morphology) thereby increasing tumour aggressiveness 53. Furthermore, PD-L1 facilitates EMT via the AKT-mTOR signalling pathway 53, a phenomenon observed across various forms of OSCC 54. Complementary to these findings, Eichberger et al. also identified that PD-L1 might regulate the activity of a specific subset of Rho-GTPases, influencing cytoskeletal organisation and potentially contributing to tumour aggressiveness 54.

Palmitoylation of PD-L1 within its cytoplasmic domain is another significant modification that enhances the stability of PD-L1 by averting ubiquitination and subsequent lysosomal degradation 55. While current studies on PD-L1 palmitoylation have mainly focused on its implications for immune function, the details of these investigations are covered in the following section.

### 2.7. ER

Oestrogen receptor (ER) is a crucial protein involved in oestrogen-mediated processes that affect reproductive health and various physiological functions in women. Immunohistochemical analyses have revealed the presence of ERβ in OSCC cell lines, with its expression levels correlating with poor prognosis, indicating its potential role as a prognostic marker in OSCC 56. Additionally, research has highlighted elevated expression of ERα in laryngeal squamous cell carcinoma compared with normal tissues 57, suggesting distinct patterns of ER expression within the different subtypes of head and neck cancer.

Palmitoylation facilitates the localisation of ERα to the plasma membrane and its interaction with caveolin-1. Upon stimulation with 17β-oestradiol (E2), palmitoylated ERα activates rapid signalling cascades implicated in cell proliferation (Figure 2C), including ERK and AKT activation, cyclin D1 promoter activity, and DNA synthesis 58. Similarly, palmitoylation is essential for the plasma membrane localisation of ERβ and its association with caveolin-1 (Figure 2D). However, in contrast to ERα, E2 binding enhances the association of ERβ with caveolin-1 and the p38 MAPK family member, leading to the inhibition of cell proliferation 59. Notably, inhibition of ERβ palmitoylation using non-specific palmitoyltransferase inhibitors, tunicamycin or 2-bromopalmitate (2-BP), disrupts the ability of the ERβ-E2 complex to activate p38, thereby impairing downstream pro-apoptotic signalling pathways, including caspase-3 activation and poly (ADP-ribose) polymerase cleavage 59.

This finding contradicts the notion of ERβ overexpression in OSCC, suggesting the intricate nature of its involvement in cancer pathogenesis. Evidence suggests that ERβ undergoes palmitoylation at cysteine 399, with ZDHHC-7 and ZDHHC-21 identified as key enzymes involved in ERα and ERβ palmitoylation 60.

Future research endeavours should focus on delineating the function of ERβ palmitoylation in modulating signalling pathways across various hierarchies and exploring its effect on OSCC development, thereby offering critical insights into prospective therapeutic targets.

### 2.9. GP130

Glycoprotein 130 (GP130) is a transmembrane protein that frequently serves as a component of the interleukin (IL) receptor family and plays a crucial role in cytokine signalling. Hyperactivation/overexpression of the IL-6/IL-6R/GP130 complex induces vimentin expression via JAK-STAT3-SNAIL signalling while suppressing E-cadherin (Figure 2E), thereby triggering EMT and enhancing tumour cell motility, which promotes metastasis of OSCC cells 61.

Palmitoylation critically regulates GP130 functionality by stabilising its membrane localisation, which is essential for downstream JAK-STAT3 signalling. This post-translational modification enhances GP130's membrane residency and amplifies its capacity to activate STAT3 phosphorylation, with pathway activity directly correlating to GP130's palmitoylation status 62, 63. Mechanistic studies have identified specific enzymes governing this process: ZDHHC5 and ZDHHC8 were shown to mediate GP130 palmitoylation in dorsal root ganglion neurons, as their reduced expression diminished GP130 surface retention 62. Similarly, ZDHHC15 deficiency was found to impair GP130 palmitoylation in glioblastoma models 63.

These observations suggest that the enzymatic activities of ZDHHC5, ZDHHC8, and ZDHHC15 are integral to the regulatory mechanisms governing GP130 localisation and function and are potential targets for therapeutic interventions aimed at modulating GP130 palmitoylation in disease.

### 2.10. Integrin α6β4

Integrin α6β4 functions as a cellular adhesion molecule, serving as receptors for the extracellular matrix, with a specific role in selecting cellular adhesion molecules 64. The overexpression of integrin α6β4 in OSCC has been identified as a prognostic marker correlated with early disease relapse and a decrease in patient survival rates 65. At the cellular membrane, integrins facilitate EGF-mediated mitogenic signalling through the EGFR and contribute to ERK signalling mediated by phosphorylated Src Family Kinases (pSFK) 66. This signalling cascade promotes cell proliferation and contributes to OSCC pathogenesis.

Early research has identified ZDHHC3 as a critical enzyme responsible for the palmitoylation of integrin α6β4 67. Genetic suppression of ZDHHC3 substantially decreased integrin α6β4 palmitoylation levels, leading to two key consequences: impaired α6 integrin-dependent cell cable formation and diminished β4 subunit phosphorylation at residues critical for hemidesmosome disassembly - a process fundamental to EMT initiation. Concurrently, impaired α6β4 signalling due to reduced palmitoylation reduces antibody-triggered Src activation, as evidenced by diminished phosphorylation levels 67. Crucially, Src serves as a bridging kinase that amplifies EGFR signalling by phosphorylating EGFR tyrosine residues, thereby enabling full receptor activation 68. The attenuated Src-EGFR crosstalk disrupts downstream ERK pathway activation - a key mitogenic driver in OSCC 67-69.

### 2.11. DSG2

Desmoglein 2 (DSG2) is a transmembrane glycoprotein characterised by its calcium-binding capacity and is implicated in cellular functions such as proliferation, survival, adhesion, and invasion in oncogenic contexts 70. Its utility as a biomarker has gained prominence owing to its significantly elevated expression levels detected in various malignancies, including squamous cell carcinoma of the lung, head and neck adenocarcinomas, and pancreatic adenocarcinomas. It has been demonstrated that DSG2 is overexpressed in OSCC 71.

Loss of DSG2 palmitoylation leads to marked downregulation of DSG2 levels by impairing its transport to the plasma membrane and promoting degradation of non-palmitoylated DSG2 72. This depletion mechanism contrasts with the pathological consequences of DSG2 overexpression in OSCC, where sustained activation of Wnt/β-catenin and PI3K-Akt signalling pathways accelerates cell cycle progression and disease malignancy 73. Meanwhile, palmitoylation enables DSG2 to coordinate endosomal protein distribution essential for lipid raft formation and small extracellular vesicle biogenesis while concurrently maintaining desmosomal protein turnover and intercellular adhesion integrity 74. Furthermore, DSG2 has been linked to an escalated production of oncogenic exosomes in the serum, stimulating fibroblast growth, augmenting the mitotic competencies of exosomes, and promoting the oncogenesis of squamous cells 74.

Investigations have elucidated that the mutations in two cysteine residues, C635 and C637, within the near-membrane structural domain of DSG2 abrogate its palmitoylation, thereby substantiating the hypothesis that these residues are critical palmitoylation sites 72.

### 2.12. PPT1

PPT1 is a lysosomal enzyme that catalyzes the protein depalmitoylation. It is considered to play a crucial role in regulating lysosomes, mitochondria and lipid metabolism 75, 76. Research indicates that PPT1 effectively promotes the proliferation, migration and invasion OSCC cells and increased PPT1 expression is found to be correlated with poor prognosis of patients. Concurrently, PPT1 influences the expression of glutathione peroxidase 4, thereby inhibiting the ferroptosis of OSCC cells 77. Furthermore, erianin, which has undergone investigation in a diverse range of human cancer cells regarding its antitumor activity, impedes growth by downregulating PPT1 in OSCC cells 78. However, it has not been demonstrated how PPT1, as a depalmitoylase, affects OSCC by modulating the palmitoylation of specific proteins.

## 3. Protein Palmitoylation and Immunotherapy in OSCC

### 3.1. PD-1&PD-L1

PD-1 and its ligand PD-L1 constitute a pair of negative immunosuppressive molecules that perform critical functions in maintaining the equilibrium between T-cell activation, immune tolerance, and progression of pathological conditions 51, 79. Located predominantly on the surfaces of macrophages, as well as on activated T and B lymphocytes, PD-1 plays a pivotal role in immune responses 51. PD-L1, which is expressed on tumours and antigen-presenting cells, binds to PD-1 on the surface of tumour-infiltrating lymphocytes, thereby exerting an inhibitory effect on lymphocyte function and enabling immune evasion by the tumour 51, 80. Based on the above findings, therapeutic antibodies against PD-L1 (e.g., atezolizumab, avelumab, and durvalumab) and PD-1 (e.g., nivolumab, pembrolizumab, and cemiplimab) were developed and have demonstrated promising results in clinical trials for various types of cancer 81. However, the effectiveness of anti-PD-1/PD-L1 treatment in OSCC remains limited, with less than 30% of patients experiencing prognostic improvements 82. This indicated the need of combining other treatments to enhance the efficacy of OSCC immunotherapy.

It has been reported that PD-L1 is palmitoylated at C272 catalysed by ZDHHC3/9 83, 84. Palmitoylation of the PD-L1 cytoplasmic domain enhances its stability by obstructing its ubiquitination and subsequent lysosomal degradation, thus preserving its presence on the cell surface and, by extension, its immunosuppressive function (Figure 3-1) 55. Notably, given the continuous trafficking of PD-L1 between the cell membrane and intracellular compartments, therapeutic antibodies demonstrate limited efficacy as they primarily target transiently surface-exposed PD-L1. In contrast, pharmacological targeting of PD-L1 palmitoylation achieves dual therapeutic effects: it not only reduces cell surface PD-L1 expression but also depletes its intracellular reservoir within recycling endosomal compartments 85. Interference with PD-L1 palmitoylation has demonstrated the potential to amplify the efficacy of immunotherapeutic strategies 4. One such approach involves the use of 2-BP, although the specificity of 2-BP for the inhibition of ZDHHCs is limited 86. Hence, the development of novel therapeutics that selectively inhibit the activity of ZDHHC3/9 could provide a precise means of modulating PD-L1 palmitoylation and warrant further research. Yao et al. engineered a peptide derived from the amino acid sequence surrounding the C272 site of PD-L1, which competitively inhibited the palmitoylation of endogenous PD-L1 by ZDHHC3, thereby diminishing PD-L1 expression in tumour cells and enhancing T-cell-mediated antitumour immunity 55. In addition, a study developed cell-penetrating peptide-induced chimera conjugates to degrade ZDHHC3, thereby directly associating ZDHHC3-mediated PD-L1 palmitoylation with the stability of PD-L1 on tumour cells 87. The chimeric conjugates demonstrated consistent therapeutic potential across experimental models: it first effectively reduced PD-L1 levels in tumour cell lines, including those with high baseline PD-L1 expression and others resistant to immune checkpoint inhibitors. Crucially, this cellular efficacy translated into potent anti-tumour activity in vivo, with no significant toxicity observed 87. These insights provide new avenues for countering PD-L1-mediated immune evasion in OSCC.

### 3.2. FAS/FASL

FAS (CD95/APO-1) is an integral member of the tumour necrosis factor receptor family and functions as a death receptor that engages with its cognate ligand FASL to initiate apoptosis. In addition to its role in cell death, FAS has been implicated in activating non-apoptotic signalling pathways such as JNK, MAPKs, and NF-κB, which can result in cell survival, proliferation, and migration 88. One retrospective study suggests that in the context of recurrent or metastatic head and neck squamous cell carcinoma, the expression level of FAS in patients exhibiting a partial response to immune checkpoint inhibitors Pembrolizumab or Nivolumab is lower than that in patients with disease progression after above inhibitor treatment 89. This indicates that FAS is a significant factor impacting the response to immunotherapy in OSCC, and that targeting FAS may enhance patient response to immunotherapy.

Palmitoylation has been recognised as a critical factor in optimising FAS-mediated cell death signalling because of its involvement in the proper localisation of FAS within cholesterol and sphingolipid-rich membrane nanodomains 90. The ability of FAS to propagate apoptotic signals through internalisation depends on the formation of supramolecular FAS aggregates, facilitated by palmitoylation of the cysteine residue at position 199 near the membrane 91. Rossin et al. identified ZDHHC7 as the principal FAS palmitoyltransferase and demonstrated that palmitoylation mediated by ZDHHC7 protects FAS from lysosomal degradation (Figure 3-2), thereby modulating its expression 90. Therefore, the targeted modulation of ZDHHC7 expression can influence FAS expression and susceptibility to apoptosis in CRC cells 90.

### 3.3. IFNGR1

Interferon-gamma (IFN-γ), predominantly produced by natural killer and T cells, exhibits context-dependent immunoregulatory effects in malignancies. IFN-γ triggers the transcription of key anti-tumour genes, including major histocompatibility complex class I, FAS, CASPASE-1, and growth-inhibitory genes, via IFNGR1-mediated JAK-STAT signalling, thereby enhancing immune cell activation and tumour cell apoptosis 92.

Recent studies have elucidated the post-translational modification of IFNGR1 via S-palmitoylation of Cys122. This modification directs the receptor towards lysosomal degradation by sorting via the adaptor protein adaptor-related protein complex 3 subunit delta 1 (AP3D1), reducing IFNGR1 expression and stability, which attenuates downstream IFN-γ signalling. Notably, palmitoylation of IFNGR1 modifies its interaction with AP3D1, potentially facilitating tumour immune evasion (Figure 3-3) in CRC 93. Critically, Porphyromonas gingivalis infection - a microbial driver shared by both oesophageal squamous cell carcinoma (ESCC) and OSCC 94 - promotes IFNGR1 palmitoylation, accelerating its lysosomal degradation and malignant progression in ESCC 95. Given the shared histological features of squamous cell carcinomas and the established role of P. gingivalis in OSCC pathogenesis, this mechanism likely extends to OSCC, where microbial-induced palmitoylation may similarly disrupt IFN-γ-mediated immune surveillance. This has critical implications for improving anti-PD-L1 immunotherapy efficacy. Yuan et al. demonstrated that high IFNGR1 expression significantly enhances anti-PD-L1 efficacy 96, suggesting that targeting IFNGR1 palmitoylation to modulate its expression may represent a promising strategy to enhance immunotherapy outcomes in OSCC. Furthermore, in addition to acting as a receptor for IFN-γ, IFNGR1 can also act as a ligand to directly activate downstream pathways. Han et al. found that OSCC-derived extracellular vesicles deliver IFNGR1 to regional lymph node stromal cells, where it activates JAK-STAT signalling independent of IFN-γ. This intercellular communication induces PD-L1 overexpression in stromal cells, facilitating CD8^+^ T-cell depletion and subsequent lymph node metastasis 97. Modulating IFNGR1 palmitoylation levels in tumour tissues may reduce "adaptive resistance" caused by OSCC-derived extracellular vesicles deliver IFNGR1.

## 4. Current Challenges and Future Perspectives

This review suggests that palmitoylation could provide a new perspective for the treatment of OSCC, although many challenges remain to be addressed. At the genetic level, it is crucial to further investigate the aberrant expression and dysregulation of palmitoylation-associated genes in OSCC and to study their correlation with survival prognosis. This exploration is pivotal for laying the groundwork for developing potential regulatory small molecules aimed at modulating palmitoylation levels through gene expression regulation, with the dual aim of controlling tumour progression and enhancing the efficacy of therapeutic interventions.

Given the variability in the characteristics of protein palmitoylation across different types of cancer, including the specific sites, magnitude, functional importance, and regulatory enzymes involved, it is evident that the phenomenon of palmitoylation, despite being extensively studied in various cancers, necessitates a more detailed investigation within the context of OSCC. This includes the need for comprehensive information regarding protein expression levels, critical signalling pathways, and, particularly, the nuanced roles of palmitoylated proteins specific to OSCC. Understanding the expression, regulatory mechanisms, enzymes involved in palmitoylation, and the associated upstream and downstream pathways, along with the unique contributions of these proteins to OSCC, presents an intriguing avenue for research.

Furthermore, beyond the well-known presence of palmitoylated proteins in tumours and immune cells, where they serve as molecular targets or modulate immune checkpoints, exploring the presence and effect of palmitoylation in other cellular types within the tumour microenvironment, such as endothelial cells and fibroblasts, holds merit. This exploration may uncover additional mechanisms by which palmitoylation influences tumour progression and the immune landscape.

Importantly, with a considerable number of patients with OSCC exhibiting resistance or a suboptimal response to immunotherapies, the role and potential exploitation of palmitoylation in this context remain to be fully elucidated. Investigating whether targeting palmitoylation and modifying the palmitoylation status of certain proteins through adjuvant therapy could enhance the tumour immune microenvironment and, by extension, improve the efficacy of immunotherapy in OSCC is a valuable research direction. Such investigations could potentially lead to novel therapeutic approaches to combat drug resistance and enhance treatment efficacy in OSCC patients.

## Abbreviations

ABHD17A/B/C: proteins 17A/B/C with α/β hydrolase domain
AP3D1: adaptor-related protein complex 3 subunit delta 1
APT1/2: acyl protein thioesterase 1/2
CDKs: cyclin-dependent kinases
Cys: cysteine
DSG2: desmoglein 2
E2: 17β-oestradiol
EGFR: epidermal growth factor receptor
EMT: epithelial-to-mesenchymal transition
ER: oestrogen receptor
ERK: extracellular signal-regulated kinase
FASL: FAS ligand
GP130: glycoprotein 130
IFN-γ: interferon gamma
IFNGR1: interferon gamma receptor 1
IL: interleukin
MAPK: mitogen-activated protein kinase
OSCC: oral squamous cell carcinoma
PD-1: programmed cell death protein 1
PD-L1: programmed cell death ligand 1
PI3K: phosphoinositide 3-kinase
PPT1/2: palmitoyl-protein thioesterase 1/2
RAB: RAS-associated binding protein
SFKs: SRC family kinases
TAZ: transcriptional coactivator with PDZ-binding motif
TEAD: transcriptional enhanced associate domain-associated protein
TGF-β: transforming growth factor beta
VEGF: vascular endothelial growth factor
VEGFR: vascular endothelial growth factor receptor
YAP: Yes-associated protein
ZDHHC: zinc finger DHHC-type containing
